# Performance of Handmade Ultra-Lightweight Hanji Treated with Wet-Strength Agents for Paper-Based Cultural Heritage Conservation

**DOI:** 10.3390/ma19183986

**Published:** 2026-09-19

**Authors:** Sang-Hyun Lee, Tae-Ho Choi

**Affiliations:** 1 Conservation and Restoration Center of Paper Heritage, Chungbuk National University, Cheongju 28644, Republic of Korea; 2 Department of Wood and Paper Science, College of Agriculture, Life & Environment Science, Chungbuk National University, Cheongju 28644, Republic of Korea; tchoi@cbnu.ac.kr

**Keywords:** Hanji, Korean traditional paper, ultra-lightweight paper, conservation, cultural heritage, wet-strength agents

## Abstract

Ultra-lightweight paper is indispensable for conserving paper-based cultural heritage. However, machine-made thin paper (MTP) exhibits pronounced strength anisotropy caused by preferential fiber orientation. This study evaluated handmade ultra-lightweight Hanji (7.2 g/m^2^) produced from *Broussonetia kazinoki* bast fiber pulp cooked with traditional plant-ash lye and formed by the *oebal* method, as a potential alternative to MTP (7.0 g/m^2^) in paper conservation. Polyamideamine-epichlorohydrin (PAE) and glyoxylated polyacrylamide (GPAM) were applied as wet-strength agents, either alone or combined with carboxymethyl cellulose (CMC). The treated papers were characterized based on weight increase, dry and wet tensile index, pH, color change, and stability after accelerated aging at 80 °C and 65% relative humidity for 72 h. PAE treatment resulted in greater weight uptake than GPAM treatment, while CMC co-treatment further increased weight uptake and tensile strength for both wet-strength agents. Unlike MTP, whose cross-directional strength could not be measured, Hanji retained comparable tensile strength in both directions, reflecting its isotropic fiber network. After aging, GPAM-treated Hanji maintained a higher pH than PAE-treated Hanji and showed less color change, whereas CMC improved strength but reduced color stability. GPAM-treated handmade Hanji provides a more pH-favorable, direction-independent, and potential alternative to MTP for paper-based cultural heritage conservation.

## 1. Introduction

Hanji, a traditional handmade Korean paper, is produced from the bast fibers of *Broussonetia kazinoki.* These fibers are characterized by long fiber length and high molecular weight, which contribute to the excellent tensile strength, tear resistance, and durability of Hanji. Because of these properties, Hanji has long been recognized as an important material for the conservation of paper-based cultural heritage [1,2].

In the conservation of paper-based cultural heritage, reinforcement paper should be chemically stable, lignin-free, pH-neutral, and composed of long fibers that can help improve the mechanical stability of the original artifact [3]. Ultra-lightweight Hanji is particularly suitable for conservation applications given its neutral pH, low thickness, high porosity, and favorable mechanical properties.

At present, machine-made conservation papers with grammages of 5, 10, 20, and 30 g/m^2^ are produced for conservation treatments, and the 5 and 10 g/m^2^ variants are commonly used for the repair of text-bearing areas [4]. Japanese thin paper [5] made from long fibers has become a primary conservation material for document preservation in Western countries given its high durability, flexibility, and excellent aging stability [6].

In conservation treatments that require moisture, thin paper can be difficult to handle because of its low grammage. In machine-made thin paper (MTP), fiber orientation is predominantly in the machine direction (MD), which can produce pronounced direction-dependent differences in mechanical strength. In contrast, handmade Hanji, the traditional Korean paper, has a more cross-oriented fiber network, resulting in minimized directional differences in strength and providing excellent dimensional stability [7].

Previous studies have investigated quality standards for using Hanji as a conservation paper with high dimensional stability [8] and methods for improving the strength of starch-treated Hanji [9]. However, research on improving the wet strength of handmade ultra-lightweight Hanji remains limited.

In the paper industry, various studies have examined methods for improving wet strength [10,11,12]. In addition, cellulose derivatives have been widely investigated for the conservation and restoration of cultural heritage materials [13,14,15,16,17]. However, only few studies have focused specifically on handmade ultra-lightweight Hanji as a traditional Korean paper.

Wet-strength agents commonly used to improve paper strength under wet conditions include polyamideamine-epichlorohydrin (PAE), melamine and urea-formaldehyde, glyoxylated polyacrylamide (GPAM), and polyvinyl alcohol (PVA).

Previous studies have reported that the combined addition of PAE and PVA significantly improves mechanical strength and surface properties compared with the addition of either agent alone [18,19]. The effects of process variables such as pressing temperature and filler addition, on wet-strength performance have also been examined [20].

Carboxymethyl cellulose (CMC) also enhances the wet strength of paper when combined with PAE [21,22]. In addition, studies on artificially aged papers made from various pulp materials have shown that chemical reinforcement using methyl cellulose (MC), CMC, PVA, and oxidized starch can improve paper stability. Among the tested application methods, cellulose derivative-based immersion and coating treatments provided the most stable reinforcement effects [23].

During the conservation and reinforcement of paper artifacts, adequate wet strength is essential for handling the paper safely. PAE, GPAM, and cellulose derivatives have been studied extensively for machine-made papers in the paper industry. However, their behavior on handmade ultra-lightweight Hanji—which has a distinct fiber morphology, an isotropic laid/chain structure, and a low grammage produced by the traditional *oebal* method—has not been established. In particular, it remains unclear how the choice of wet-strength agent (PAE vs. GPAM) and its combination with CMC affects not only the wet strength but also the pH and aging stability of such ultra-lightweight Hanji.

Therefore, this study investigates two questions: (i) whether PAE and GPAM, alone or in combination with CMC, can impart sufficient wet strength to handmade ultra-lightweight Hanji for conservation handling; and (ii) how these treatments differ in their effects on pH and accelerated-aging stability. The novelty of this study lies in applying and systematically comparing these wet-strength systems on traditionally produced ultra-lightweight Hanji, and in evaluating its potential as an alternative to MTP from an application perspective.

In this study, handmade ultra-lightweight Hanji with a grammage of 7.2 g/m^2^ was produced from *Broussonetia kazinoki* bast fiber pulp cooked using traditional plant-ash lye obtained from burning red pepper stems. The wet-strength agents PAE and GPAM were applied alone and in combination with CMC, and the physical properties, pH, and accelerated-aging stability of the treated Hanji were compared with those of MTP.

## 2. Materials and Methods

### 2.1. Raw Materials

The handmade ultra-lightweight Hanji used in this study was produced by *Mungyeong Hanji* using traditional methods. As a control, an MTP (commercial name: *Tengujo*) was purchased in 2024 from a specialty washi retail store in Kyoto, Japan. The manufacturer was neither identified on the product label nor available from the retailer. The characteristics of the handmade ultra-lightweight Hanji and MTP are summarized in Table 1.

To improve wet strength, PAE and GPAM were used, and CMC was used as a cellulose derivative for strength enhancement. The characteristics of each are summarized in Table 2.

### 2.2. Preparation of Ultra-Lightweight Hanji

Handmade ultra-lightweight Hanji was prepared from the white inner bark of one-year-old *Broussonetia kazinoki.* The bark was cooked at 100 °C for 2 h using traditional plant-ash lye prepared from red pepper stems. After cooking, the *Broussonetia kazinoki* pulp was mixed with water at 15 °C and *Abelmoschus manihot* mucilage with a viscosity of 30 cP. The pulp suspension was dispersed in a wooden vat measuring 200 × 240 × 50 cm and formed into sheets using a traditional *oebal*. A total of 100 wet sheets were then pressed using a hydraulic press and dewatered at a pressure of 36 kPa for 8 h, after which they were attached to drying boards and dried to produce Hanji.

Each of the 100 sheets was formed individually as an independent handmade sheet. Because the grammage of handmade sheets varied slightly, sheets with a grammage close to 7.0 g/m^2^ were selected to ensure uniformity (mean grammage of 7.2 g/m^2^). The selected sheets were then randomly assigned to the different treatment groups (untreated, PAE, GPAM, PAE + CMC, and GPAM + CMC).

### 2.3. Application of Wet-Strength Agents

The handmade ultra-lightweight Hanji and MTP were cut to 20 × 20 cm specimens. The handmade ultra-lightweight Hanji was evaluated in the laid direction (LD) and chain direction (CD), whereas the MTP was evaluated in the machine direction (MD) and cross direction (CD).

Wet-strength agent solutions were prepared by dissolving PAE, GPAM, and CMC in distilled water to a concentration of 0.5% based on solid content. For the combined treatments, PAE (or GPAM) and CMC were each prepared as separate 0.5% (*w*/*w*, based on solid content) solutions and mixed at a 1:1 volume ratio immediately before spraying, resulting in a final applied concentration of 0.25% PAE (or GPAM) and 0.25% CMC. The viscosity of each additive solution was measured at 25 °C using a Brookfield viscometer (Model DV-II +Pro, Brookfield, Middleboro, MA, USA) with spindle number 61 and operated at 200 rpm.

Each 20 × 20 cm specimen was placed on an aluminum plate, and the 0.5% wet-strength agent solution was sprayed evenly from a distance of 1 m using a spray gun (SG 1200, MOA, Shanghai, China). Spraying was performed over three sections of the specimen, namely the top, middle, and bottom regions, for 3 s. The treated specimens were then cured at 60 °C for 2 h in a constant-temperature drying oven (DKM610C, YAMATO, Tokyo, Japan). The weights of the specimens before and after wet-strength agent treatment were measured, and the weight increase was calculated using the following equation:

(1) $$W\left(\%\right)=\frac{A-B}{B}\times 100$$

where W is the weight increase rate (%), *A* is the specimen weight after treatment (g), and *B* is the specimen weight before treatment (g).

### 2.4. Accelerated Aging of Ultra-Lightweight Paper

Accelerated aging of the handmade ultra-lightweight Hanji and MTP was performed according to ISO 5630-3 [24] using a constant-temperature and humidity chamber (ACE180, ACEONE Co., Incheon, Republic of Korea) at 80 ± 0.5 °C and 65 ± 2% relative humidity (RH) for 24, 48, and 72 h. The maximum aging duration of 72 h was selected to evaluate the short-term response of the treated ultra-lightweight papers under controlled hygrothermal conditions and to compare relative changes among treatments. This duration does not establish an equivalent period of natural aging and should not be interpreted as direct evidence of long-term conservation stability. Independent specimens were used at 0 h (unaged control) and at each aging time point (24, 48, and 72 h).

### 2.5. Properties of Ultra-Lightweight Paper

The specimens used in the experiment were conditioned for 24 h at 23 ± 1 °C and 50 ± 2% RH in accordance with KS M ISO 187 [25] before testing. The tensile index and wet tensile index were measured 10 times for each sample (*n* = 10) using a horizontal tensile strength tester (SE062, Lorentzen & Wettre, Kista, Sweden) in accordance with ISO 1924 [26] and ISO 3781 [27], and the average values were calculated.

The surface pH of the samples before and after the treatment with a wet-strength agent and before and after accelerated aging was measured in accordance with TAPPI T 529 [28] using a pH meter equipped with a flat surface electrode (Orion 3 Star, Thermo Scientific, Waltham, MA, USA). A drop of distilled water was placed on the specimen surface, and the flat electrode was brought into contact with the wetted area to record the pH.

### 2.6. Optical Properties of the Ultra-Lightweight Paper

The optical properties of the samples before and after treatment, as well as before and after accelerated aging, were measured using a colorimeter (Color-Eye 7000A, X-RITE, Grand Rapids, MI, USA) based on the CIE LAB color space (L*, a*, b* values). The smaller the value of the color difference (ΔE) is, the less the color changed in the paper sample before and after accelerated aging. The color difference (ΔE) in the CIE L*a*b* color system was calculated as follows (2):

(2) $$\Delta E=\sqrt{{\left(\Delta L*\right)}^{2}+{\left(\Delta a*\right)}^{2}+{\left(\Delta b*\right)}^{2}}$$

where ΔE is the total color difference, ΔL* is the difference in lightness, Δa* is the difference along the red–green axis, and Δb* represents the difference in the yellow–blue axis.

### 2.7. Scanning Electron Microscopy and Portable Fourier-Transform Infrared Spectroscopy

Untreated paper sheets and paper sheets treated with PAE, GPAM, and CMC were analyzed using a portable Fourier-transform infrared (FT-IR) spectrometer (4300 Handheld FTIR, Agilent, Santa Clara, CA, USA) equipped with a diamond-attenuated total reflection (ATR) probe. Spectra were collected over the range of 4000–650 cm^−1^ at a spectral resolution of 8 cm^−1^, and each spectrum was obtained by averaging 32 scans. The transmittance spectra were converted to absorbance using A = 2 − log_10_(%T).

The surfaces of the untreated and treated paper samples were observed using scanning electron microscopy (SEM; Gemini 560, Zeiss, Oberkochen, Germany) at an accelerating voltage of 3 kV and a working distance of 4.5 mm. Before imaging, the samples were coated twice with Pt using a sputter coater (150T ES plus, Quorum, Laughton, UK).

FT-IR and SEM analyses were performed on one representative specimen per treatment condition, selected as representative of the batch used for the quantitative measurements. These analyses were used for qualitative confirmation of chemical bonding and surface morphology rather than for statistical comparison between conditions. SEM images were acquired at multiple locations and magnifications on each specimen, and the representative images are shown.

### 2.8. Statistical Analysis

All values were expressed as mean ± standard deviation (SD). Tensile and wet-tensile measurements were obtained from 10 independently prepared sheets per condition (*n* = 10), pH from five independent specimens (*n* = 5), and color coordinates from three independent sheets (*n* = 3). The effects of wet-strength agent, aging time, and testing direction were evaluated using analysis of variance (ANOVA; three-way for tensile properties and two-way for pH), followed by Tukey’s honestly significant difference (HSD) post hoc test when a significant effect was found (*p* < 0.05). Normality and homogeneity of variance were checked using the Shapiro–Wilk and Levene tests. Analyses were performed in Python 3 (statsmodels, SciPy).

## 3. Results and Discussion

### 3.1. Overview of Statistical Analysis

Table 3 summarizes the ANOVA results for the tensile index, wet tensile index, and pH of the PAE- and GPAM-treated ultra-lightweight Hanji.

For the dry tensile index, the wet-strength agent (F(1, 143) = 503.1, *p* < 0.001), aging time (F(3, 143) = 8.1, *p* < 0.001), and testing direction (F(1, 143) = 52.7, *p* < 0.001) had significant main effects. The agent × aging time interaction was not significant (*p* = 0.306), whereas the agent × aging time × direction interaction was highly significant (F(3, 143) = 16.3, *p* < 0.001). This pattern indicates that the influence of aging on the tensile index cannot be interpreted without considering the testing direction. Tukey’s HSD test confirmed that the GPAM-treated Hanji remained statistically unchanged between 0 and 72 h in both the CD (*p* = 0.999) and LD (*p* = 0.245), whereas the PAE-treated Hanji increased significantly in the CD (*p* < 0.001) and decreased significantly in the LD (*p* < 0.001) over the same period. Because these two directional changes are opposite, they cancel each other when the directions are combined, which explains the nonsignificant agent × aging time interaction.

A similar pattern was observed for the wet tensile index, for which the agent effect (F(1, 65) = 86.2, *p* < 0.001) and the three-way interaction (F(3, 65) = 7.4, *p* < 0.001) were both significant; the superiority of GPAM in the CD became significant after 48 and 72 h of aging (*p* < 0.01). For pH, both the agent (F(1, 30) = 2617, *p* < 0.001) and aging time (F(3, 30) = 258.6, *p* < 0.001) were highly significant, and Tukey’s HSD test confirmed that the GPAM-treated Hanji maintained a significantly higher pH than the PAE-treated Hanji at every aging time (*p* < 0.001).

### 3.2. Weight Increase

**Figure 1:**
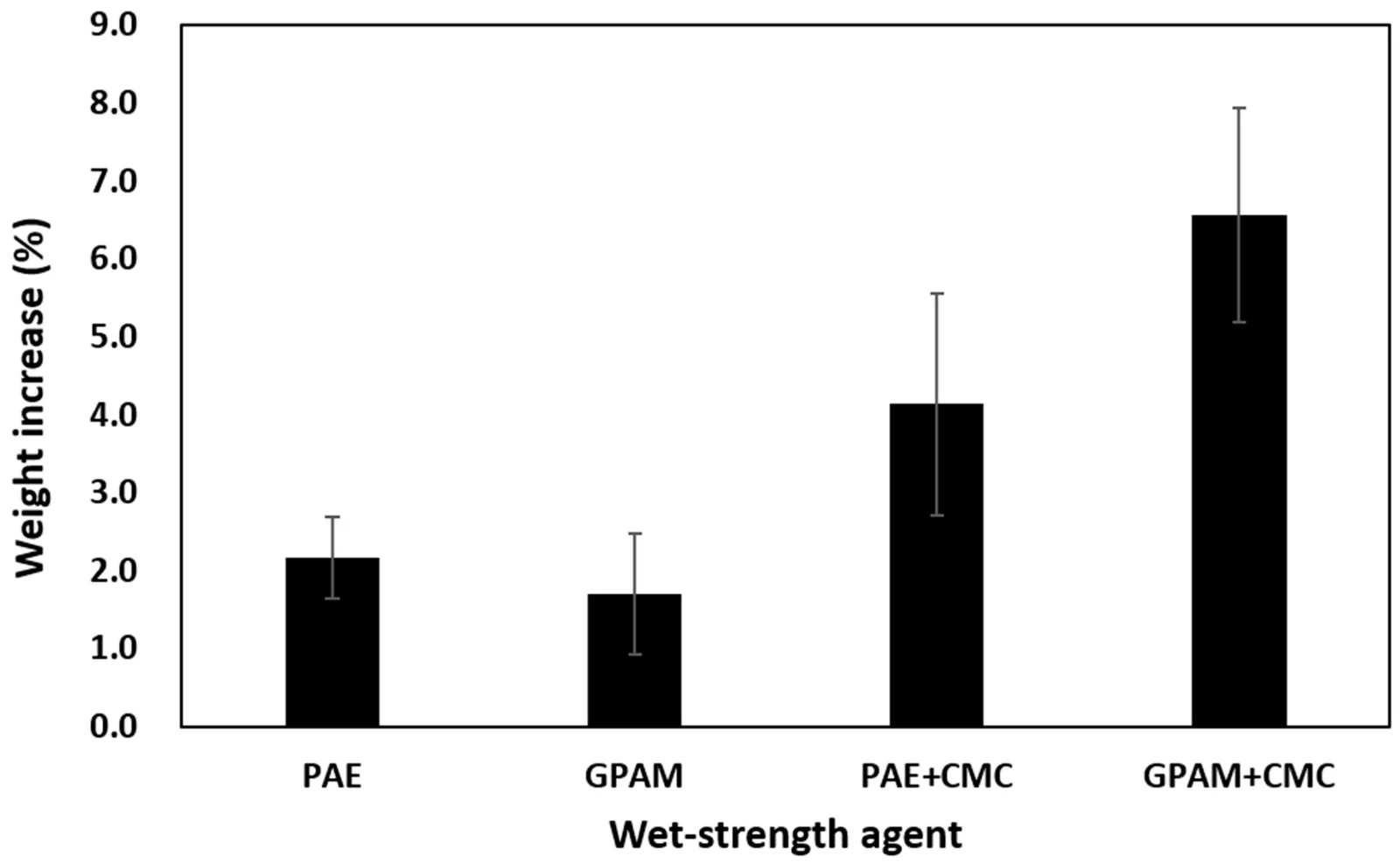
Weight increase rate of ultra-lightweight Hanji after application of wet-strength agents. Error bars represent the standard deviation (SD).

The weight increases in handmade ultra-lightweight Hanji treated with wet-strength agents are shown in Figure 1. The results showed that the weight increase was greater after treatment with PAE than that after treatment with GPAM. This difference is likely related to differences in the retention behavior of the two agents. GPAM is generally reported to form mainly hydrogen bonds with cellulose fibers, whereas PAE can additionally undergo self-crosslinking between polymer chains, which may enhance its retention on the fiber network [29].

Treatment with the cellulose derivative CMC further increased the weight uptake of ultra-lightweight Hanji. Cationic PAE resin can interact with the anionic CMC to form a weakly cationic polymer complex, often referred to as a “symplex,” which adsorbs onto the fiber surface and thereby improves chemical retention. Consequently, the carboxyl groups in CMC likely promoted the adsorption of PAE and GPAM onto the fiber surface, contributing to the increased weight uptake [21].

Based on the weight increase and the paper grammage, the amounts of PAE and GPAM deposited were approximately 0.19 ± 0.04 and 0.15 ± 0.07 g/m^2^, respectively; the corresponding values for the PAE + CMC and GPAM + CMC treatments were 0.36 ± 0.12 and 0.59 ± 0.12 g/m^2^.

### 3.3. Strength of Ultra-Lightweight Hanji Treated with Wet-Strength Agent

**Figure 2:**
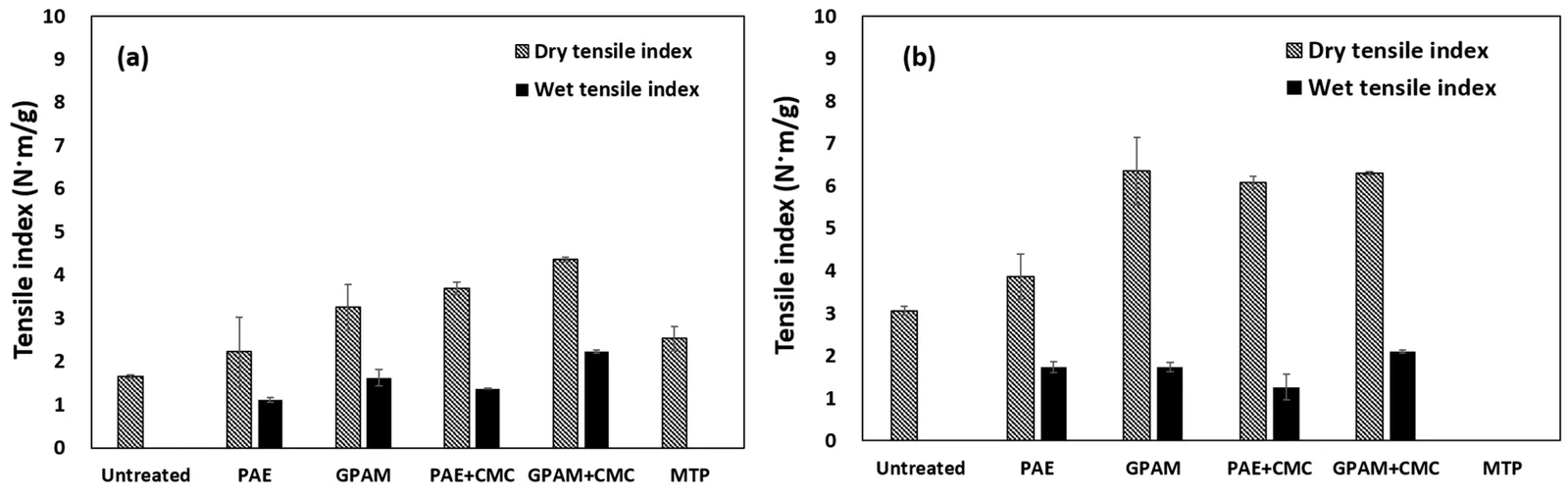
Tensile index of ultra-lightweight Hanji treated with wet-strength agents: (**a**) laid direction (LD) of Hanji and machine direction (MD) of MTP; (**b**) chain direction (CD) of Hanji and cross direction (CD) of MTP. Error bars represent the standard deviation (SD).

The dry and wet tensile index values of ultra-lightweight Hanji after treatment with wet-strength agents are shown in Figure 2. For untreated handmade ultra-lightweight Hanji, the dry tensile index could be measured in both the laid direction (LD) and chain direction (CD). By contrast, for MTP, the dry tensile index could be measured only in the machine direction (MD), whereas the cross direction (CD) tensile index could not be measured. This difference is primarily related to the fiber orientation produced by the respective papermaking methods [7]. In machine-made thin paper, the fibers are strongly aligned in the machine direction (MD); consequently, very few fibers and interfiber bonds are oriented in the cross direction (CD), resulting in extremely low CD strength. When CD measurement was attempted, the specimens tore during handling and mounting before a valid tensile value could be recorded. By contrast, the isotropic laid/chain fiber network of the handmade Hanji provided measurable tensile strength in both directions.

The differences observed between handmade ultra-lightweight Hanji and MTP should not be attributed solely to the handmade and machine-made forming methods. The materials may also differ in terms of raw material, cooking chemistry, drying history, and other structural parameters. Therefore, the observed differences in tensile behavior cannot be attributed exclusively to fiber orientation, and the comparison should be regarded as an evaluation of the two papers as complete material systems from an application perspective rather than as an isolated assessment of the forming process.

The wet tensile index of both handmade ultra-lightweight Hanji and untreated MTP could not be measured in either direction. This result indicates that moisture weakened the hydrogen bonding between fibers during wet treatment, leading to a substantial loss of mechanical integrity.

For handmade ultra-lightweight Hanji, treatment with a wet-strength agent improved tensile performance. Furthermore, CMC co-treatment further increased the tensile index compared with treatment using PAE or GPAM alone. These results indicate that wet-strength agent treatment enhanced interfiber bonding, thereby improving the mechanical strength of the ultra-lightweight Hanji under both dry and wet conditions. Three-way ANOVA confirmed that the wet-strength agent significantly affected the tensile index (*p* < 0.001; Table 3).

### 3.4. Changes in the Tensile Strength of the Ultra-Lightweight Hanji After Accelerated Aging

**Figure 3:**
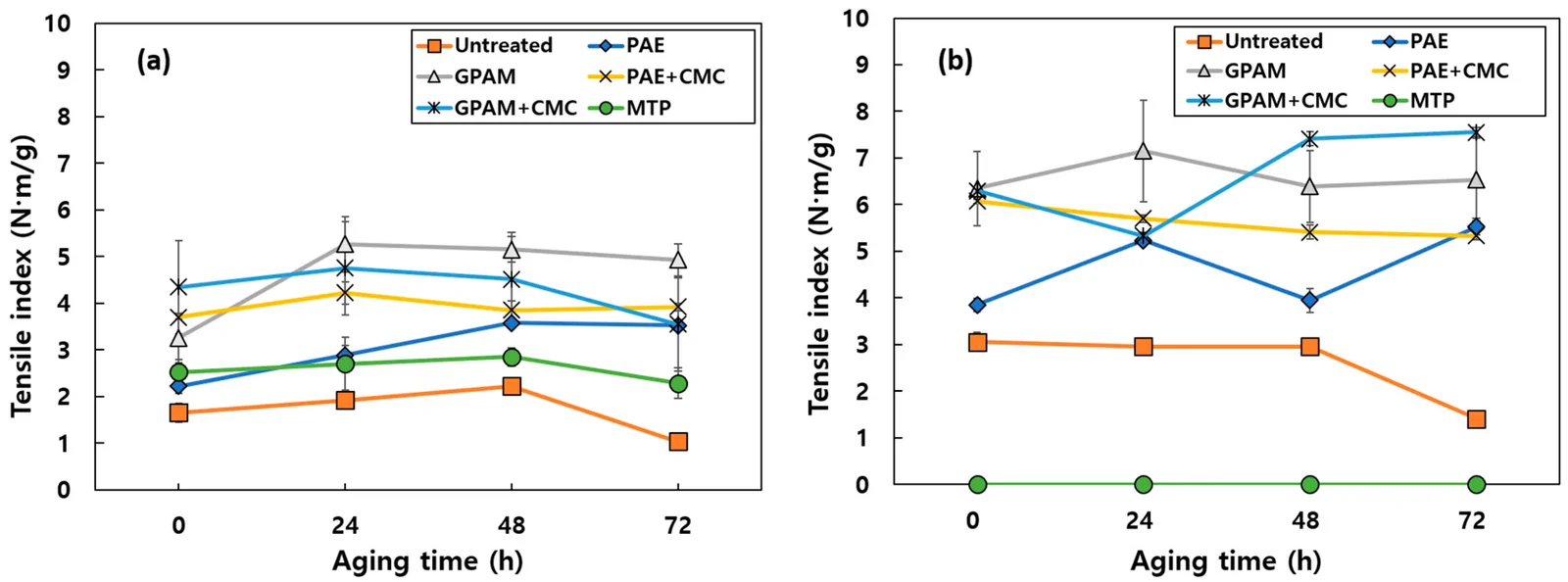
Changes in tensile index of ultra-lightweight Hanji after accelerated aging: (**a**) laid direction (LD) of Hanji and machine direction (MD) of MTP; (**b**) chain direction (CD) of Hanji and cross direction (CD) of MTP. Error bars represent the standard deviation (SD).

The changes in the dry tensile index of handmade ultra-lightweight Hanji and MTP treated with wet-strength agents after accelerated aging are shown in Figure 3. Both the LD and CD tensile indices of handmade ultra-lightweight Hanji increased when treated with the wet-strength agents PAE and GPAM. This suggests that treatment with PAE and GPAM improved the interfiber bonding strength. Hanji fibers are interlaced during the papermaking process, resulting in a paper with minimal strength difference between the LD and the CD. Meanwhile, in the case of MTP, most of the fibers are oriented in the MD, resulting in a clear difference in strength between the MD and CD [7]. Therefore, the tensile strength of MTPs made using the paper machine method could be measured in the MD but not in the CD.

For handmade ultra-lightweight Hanji treated with wet-strength agents, the tensile index after accelerated aging was higher in the CD than in the LD. Additionally, GPAM-treated Hanji showed higher tensile strength and better aging stability than PAE-treated Hanji. This difference is likely related to differences in the bonding chemistry of the two agents rather than to the relative abundance of functional groups. GPAM forms hemiacetal linkages with the hydroxyl groups of cellulose and additional hydrogen bonds through its amide groups, whereas PAE forms ester bonds via its azetidinium groups; these bond types differ in their hydrolytic and thermal stability, which may contribute to the improved retention of tensile strength and aging stability observed for GPAM-treated Hanji [29,30,31,32]. The wet-strength mechanisms of these polymers are complex, and the present results do not allow the contribution of individual bonding pathways to be isolated. The agent × aging time × direction interaction was significant (*p* < 0.001), confirming that PAE- and GPAM-treated papers aged differently depending on testing direction (Table 3).

**Figure 4:**
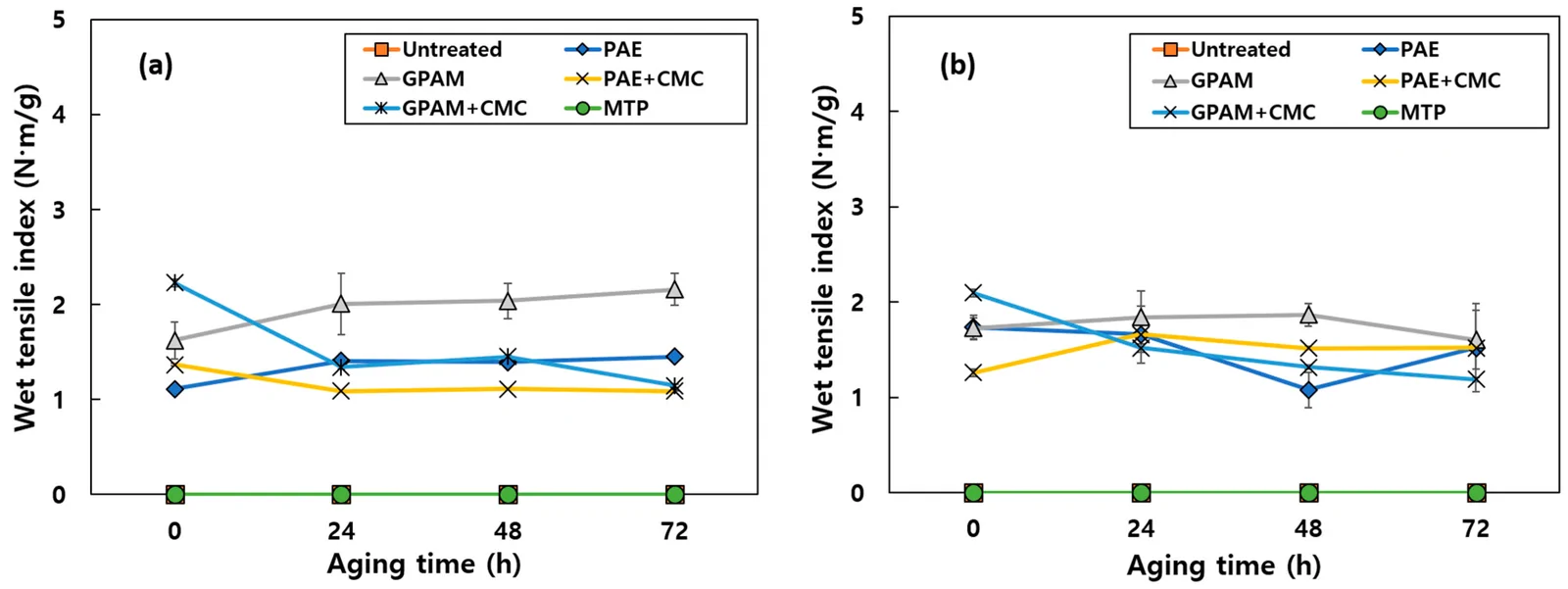
Changes in wet tensile index of ultra-lightweight Hanji after accelerated aging: (**a**) laid direction (LD) of Hanji and machine direction (MD) of MTP; (**b**) chain direction (CD) of Hanji and cross direction (CD) of MTP. Error bars represent the standard deviation (SD).

The changes in the wet tensile index after accelerated aging are shown in Figure 4. The wet tensile index of untreated handmade ultra-lightweight Hanji and MTP could not be measured. This was attributable to moisture-induced weakening of interfiber hydrogen bonding during wet treatment, which reduced the mechanical integrity of the untreated papers.

The wet tensile index of handmade ultra-lightweight Hanji treated with PAE and GPAM exceeded 15% of the corresponding dry tensile index, which is the generally accepted threshold for classifying a paper as a wet-strength paper [21]. These results indicate that both PAE and GPAM are effective wet-strength agents for handmade ultra-lightweight Hanji. Because handmade ultra-lightweight Hanji was produced using the *oebal* hand-sheeting method, it showed only minor directional differences in wet tensile index between the CD and LD. Additionally, the wet tensile index of PAE- and GPAM-treated Hanji changed only slightly with increasing aging time, indicating relatively stable performance under the applied accelerated-aging conditions.

### 3.5. Changes in pH

The pH of the untreated handmade ultra-lightweight Hanji was 6.73, which was similar to that of MTP at 6.74. Treatment with wet-strength agents generally decreased the pH of the Hanji. PAE-treated ultra-lightweight Hanji had a pH of 4.95, indicating acidic conditions, whereas PAE + CMC-treated Hanji had a pH of 5.34, indicating a slightly less acidic condition. By contrast, GPAM-treated ultra-lightweight Hanji had a pH of 6.62 and GPAM + CMC-treated Hanji had a pH of 6.59.

**Figure 5:**
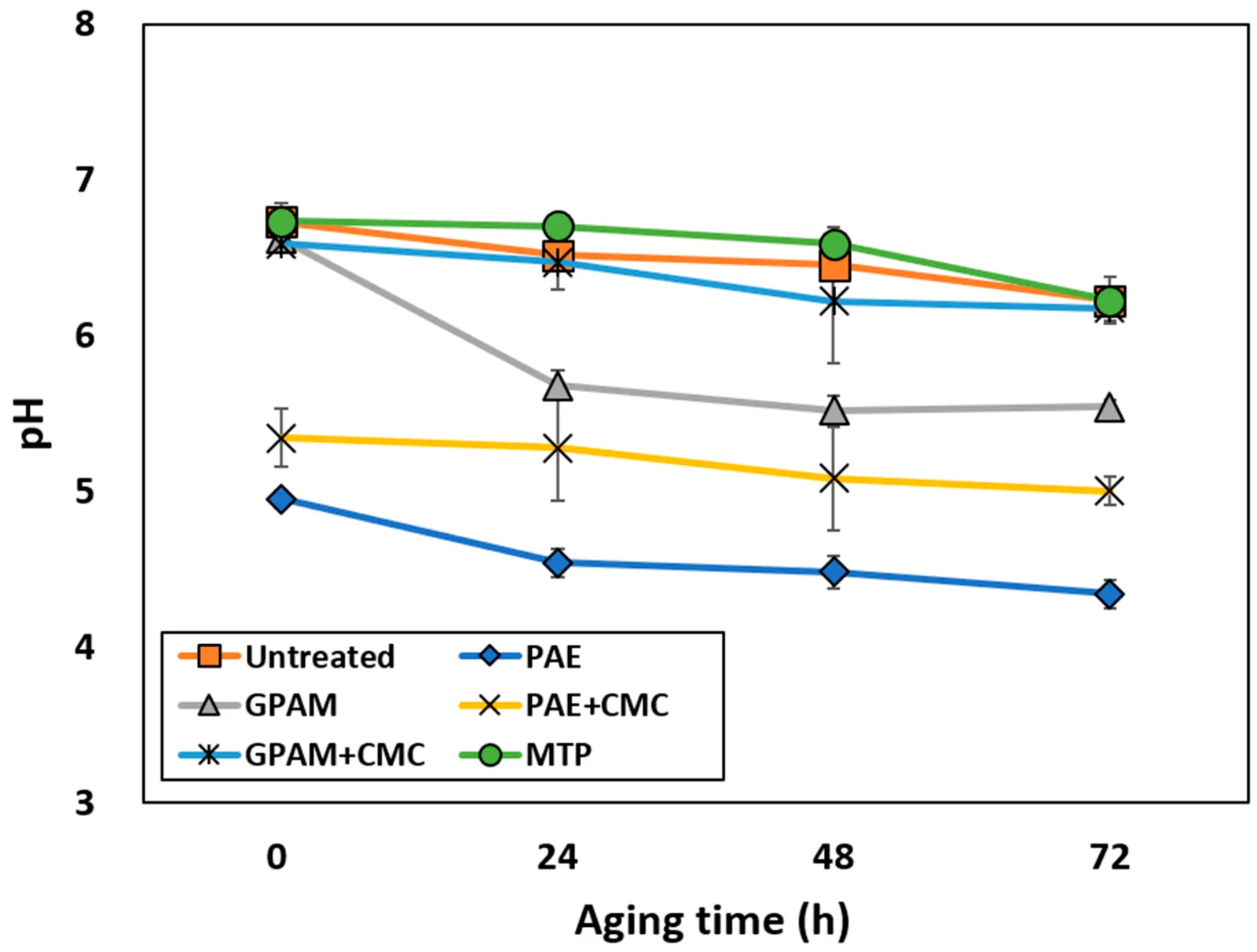
Changes in the pH of ultra-lightweight Hanji during accelerated aging. Error bars represent the standard deviation (SD).

Changes in the pH of handmade ultra-lightweight Hanji treated with different wet-strength agents during accelerated aging are shown in Figure 5. Regardless of the wet-strength agent used, the pH gradually decreased with increasing aging time.

However, GPAM-treated ultra-lightweight Hanji consistently maintained a higher pH than PAE-treated Hanji.

The decrease in the paper pH after PAE treatment may be attributed primarily to the acidic nature of commercially available PAE resins, which generally have a pH of approximately 3–5. During curing, the azetidinium groups present in the PAE resin react with the carboxyl groups of cellulose to form crosslinks. By contrast, GPAM is generally prepared and applied under near-neutral conditions and is therefore expected to have a smaller effect on paper pH [33]. Notably, the reported values are of surface pH. Although a polymer coating could partially shield the underlying fiber acidity during the 72 h accelerated aging, the consistently higher surface pH of the GPAM-treated papers, together with their smaller loss of tensile strength, is consistent with genuinely milder acid-catalyzed degradation rather than a purely transient masking effect. Longer-term aging would help distinguish a shielding effect from intrinsic pH stability.

As a less acidic pH is generally more favorable for the preservation treatment of cultural heritage materials, GPAM showed more favorable pH stability under the investigated aging conditions. The higher pH of GPAM-treated Hanji compared with that of PAE-treated Hanji was statistically significant at every aging time (*p* < 0.001; Table 3).

### 3.6. Color Changes During Accelerated Aging

To evaluate aging-induced color changes in handmade ultra-lightweight Hanji treated with different wet-strength agents, the color difference (ΔE) was calculated after measuring the L*, a*, and b* values, as shown in Table 4.

Treatment with the wet-strength agent decreased the L*, a*, and b* values of handmade ultra-lightweight Hanji, indicating a reduction in brightness, red tint, and yellow tint. The lightness (L*) of PAE + CMC and GPAM + CMC was markedly lower than that of the papers treated with PAE or GPAM alone. As the aging time increased, the L* and a* values decreased, and the b* value increased, resulting in a decrease in redness and an increase in yellowness, although the change in lightness was comparatively small. The reduction in red tint and the increase in yellow tint were greater with PAE treatment compared to those with GPAM treatment.

**Figure 6:**
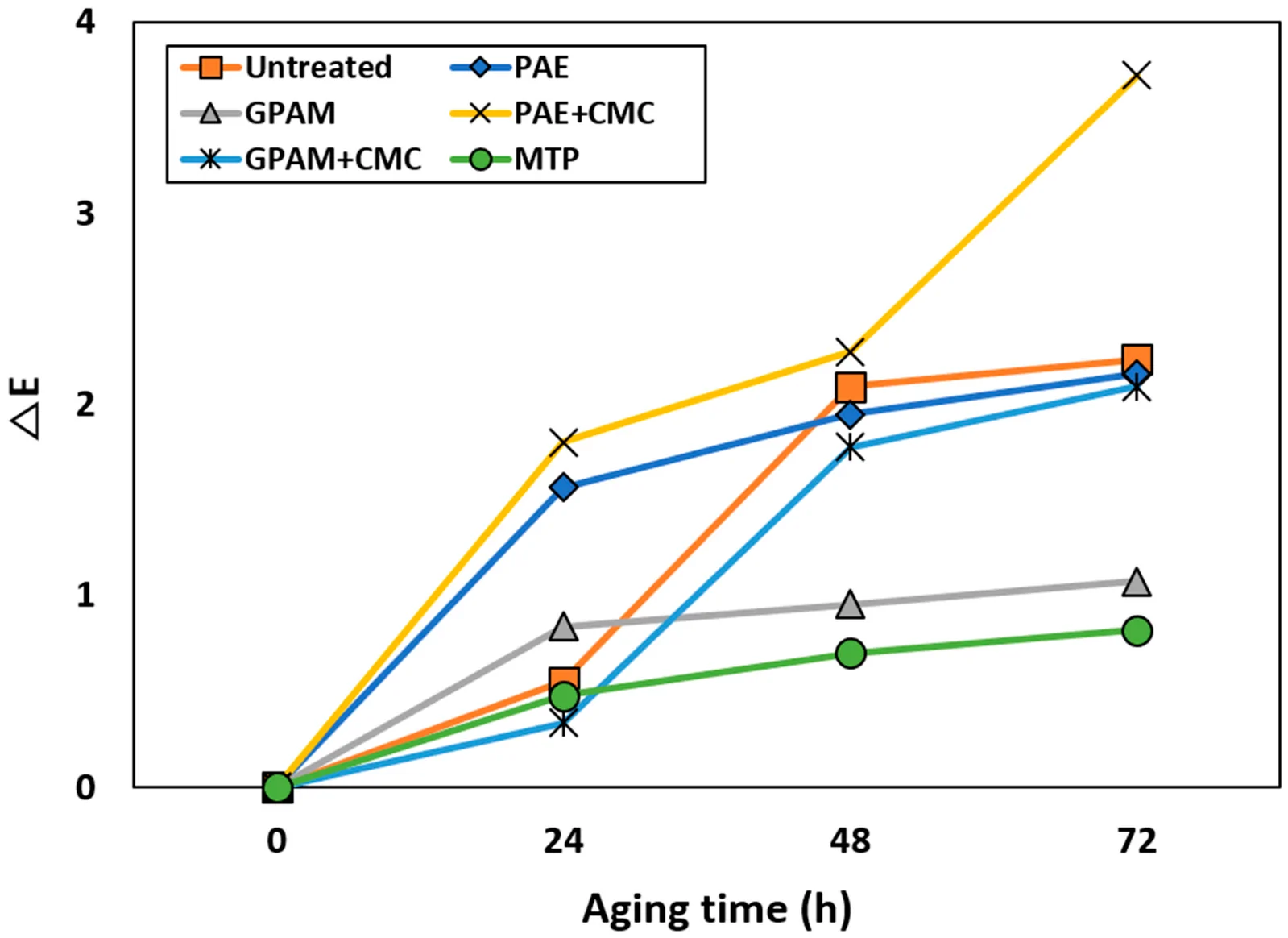
Color difference after the accelerated aging of ultra-lightweight Hanji.

The changes in color difference (ΔE) due to the deterioration of handmade ultra-lightweight Hanji treated with different wet-strength agents are shown in Figure 6. The PAE + CMC-treated sample showed the maximum color change after aging, and the MTP sample showed the minimum color change. After 72 h of aging, the color difference (ΔE) of the PAE-treated ultra-lightweight Hanji was 2.16, and the color difference (ΔE) of the GPAM-treated ultra-lightweight Hanji was 1.07. This result indicates that the PAE-treated ultra-lightweight Hanji had a more pronounced color change after aging than the GPAM-treated ultra-lightweight Hanji. Furthermore, CMC co-treatment of Hanji with a wet-strength agent improved strength but negatively affected the color stability.

A pronounced decrease in the initial (unaged) L* value was observed for the CMC-containing treatments, from approximately 80 for the untreated, PAE-, and GPAM-treated papers to 58.4 (PAE + CMC) and 57.8 (GPAM + CMC), accompanied by a decrease in b*. As all specimens were measured against the same black backing and because the ultra-lightweight Hanji is highly porous, the CMC film filled the interfiber pores and increased the translucency of the sheet, allowing the black backing to show through; this largely accounts for the low measured L* and b* of the CMC-treated papers rather than an intrinsic darkening of the paper.

In addition to this initial optical change, the CMC-containing treatments showed the largest color change (ΔE) during accelerated aging (Figure 6). The mechanism underlying this color destabilization was not experimentally isolated in this study; however, several pathways may contribute to this. As a hygroscopic polysaccharide, CMC may retain additional moisture and residual acidity at the fiber surface, promoting acid- and moisture-catalyzed degradation of cellulose and the formation of chromophores during aging. CMC may also undergo thermal and oxidative degradation, generating colored products, and may interact with residual noncellulosic components (e.g., lignin or natural colorants) in the *Broussonetia kazinoki* fibers in a manner that accelerates yellowing. To place these values in context, a color difference in ΔE ≈ 1 is generally regarded as the threshold perceptible to a trained observer, whereas ΔE > 2–3 is noticeable to the average observer [34,35]. The CMC-containing treatments reached ΔE up to 3.72 (PAE + CMC) after aging, clearly exceeding the threshold of visual perceptibility, whereas GPAM alone remained close to ΔE ≈ 1. This tradeoff indicates that CMC co-treatment should be applied with caution where preservation of the original appearance is a priority.

Both the initial increase in translucency and the greater color change during aging can alter the visual appearance of a treated artifact and therefore remain important considerations for conservation applications.

### 3.7. Fourier-Transform Infrared (FT-IR) Spectroscopy

**Figure 7:**
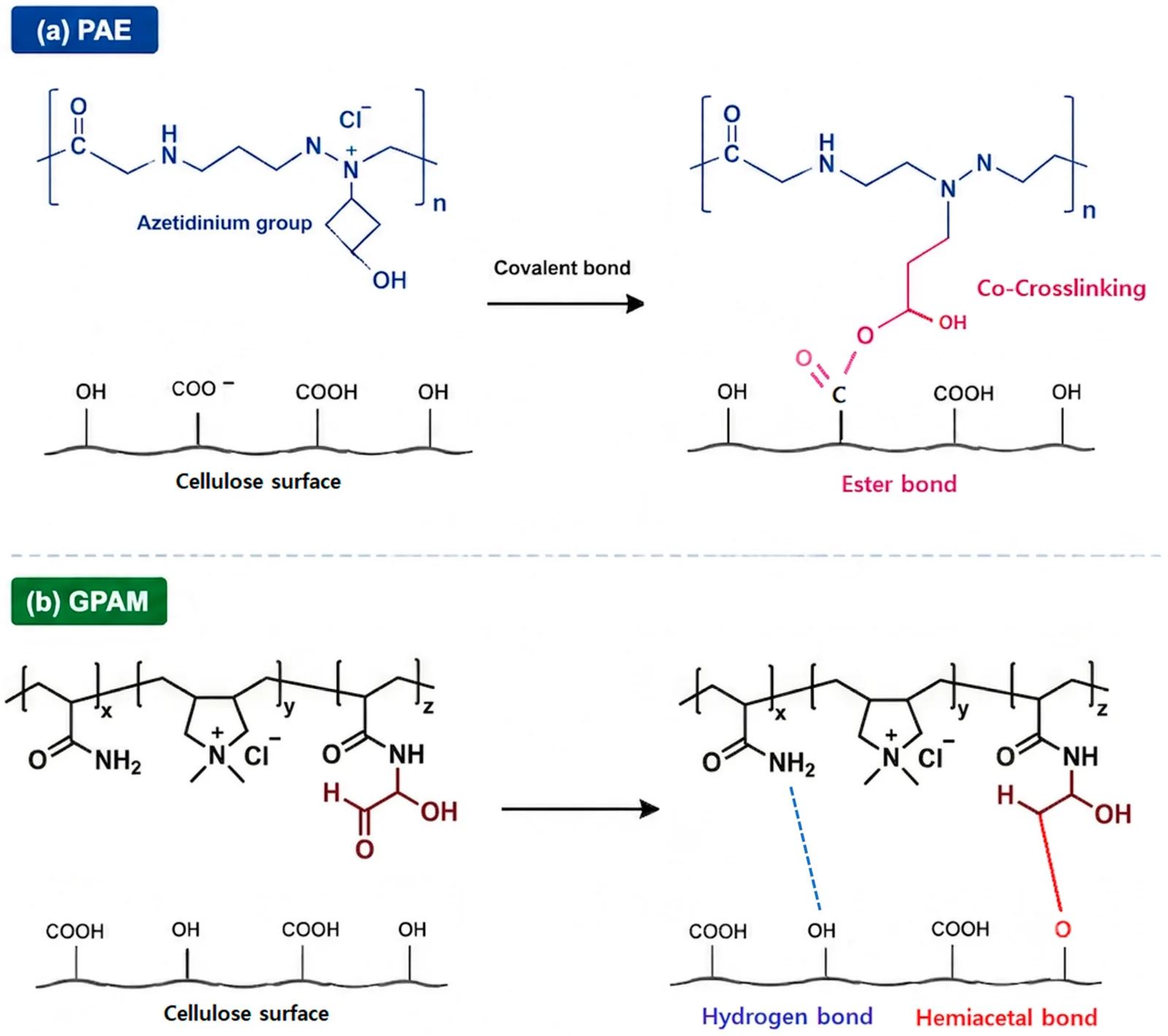
Bonding mechanisms of PAE and GPAM on cellulose surfaces. (**a**) PAE azetidinium groups react with cellulose carboxyl groups to form covalent ester bonds; (**b**) GPAM glyoxal-aldehyde groups form hemiacetal linkages with cellulose hydroxyl groups. Redrawn and modified based on a previous report [29].

Figure 7 illustrates the bonding mechanism between cellulose fibers and the wet-strength agents, PAE and GPAM used in this study.

According to previous studies, PAE provides wet strength through the formation of covalent ester bonds between the azetidinium group in PAE and carboxyl groups on cellulose, whereas GPAM forms hemiacetal linkages between glyoxal aldehyde groups and hydroxyl groups on cellulose [29]. The amide groups (-CONH_2_) in GPAM have also been reported to interact with hydroxyl groups on the fibers through hydrogen bonding, contributing to improved dry strength of the paper [29]. The covalent bonds between the wet-strength agents and the cellulose fibers are known to be water-resistant, thereby improving the mechanical strength of the wet paper [33].

**Figure 8:**
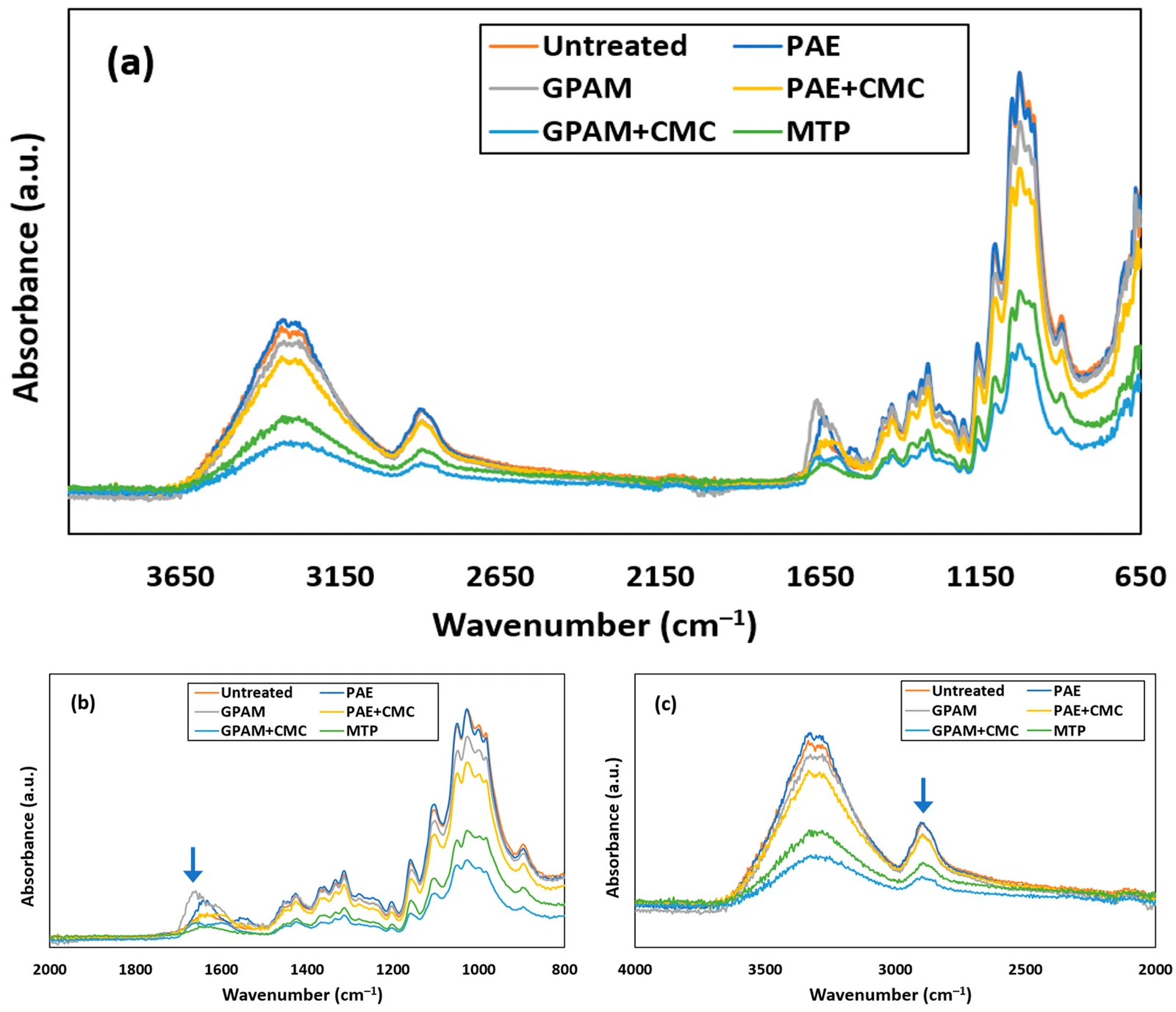
FT-IR absorbance spectra of ultra-lightweight Hanji treated with different wet-strength agents: (**a**) full-range spectra (4000–650 cm^−1^), (**b**) expanded carbonyl and fingerprint region (2000–800 cm^−1^), and (**c**) expanded hydroxyl stretching region (4000–2000 cm^−1^).

Figure 8 shows the FT-IR spectra of ultra-lightweight Hanji treated with different wet-strength agents. The full spectral range is shown in Figure 8a. Figure 8b presents the expanded carbonyl region, where absorption bands between 1735 and 1550 cm^−1^ are associated with C=O functional groups, consistent with the proposed formation of ester linkages between PAE and cellulose. Figure 8c presents the expanded high-wavenumber region, including the C–H stretching band near 2936 cm^−1^, which corresponds to methylene groups in the main chains of PAE and GPAM in the treated ultra-lightweight Hanji [31,32].

As shown in Figure 8b, the absorption intensity in the 1735–1550 cm^−1^ region corresponding to the C=O functional group of the ester bond between PAE and cellulose fibers increased after PAE treatment. Furthermore, a strong absorption band near 2900 cm^−1^ was observed and attributed to C–H stretching vibrations. These spectral changes indicate that PAE was successfully introduced into the ultra-lightweight Hanji and are consistent with the proposed formation of ester bonds between PAE azetidinium groups and cellulose carboxyl groups; by contrast, these absorption features were barely observed in the FT-IR spectrum of untreated ultra-lightweight Hanji. The FT-IR spectra also showed features associated with GPAM and CMC. In the GPAM-treated Hanji, the O–H stretching band centered near 3334 cm^−1^ in the untreated paper shifted to a lower wavenumber of approximately 3280 cm^−1^, and the absorption near 1660 cm^−1^, assignable to the amide I (C=O) vibration of the amide (–CONH_2_) groups of GPAM, increased relative to the untreated paper. The shift in the O–H band to a lower wavenumber is consistent with enhanced hydrogen bonding between the amide and hydroxyl groups of GPAM and the hydroxyl groups of cellulose. For the CMC-containing treatments, an increase in the absorption near 1600 cm^−1^, attributable to the asymmetric stretching of carboxylate (COO^−^) groups, was observed, which is consistent with the introduction of CMC onto the fibers. The remaining bands were common to all samples and were assigned to the cellulose structure, including C–H stretching near 2900 cm^−1^ and the C–O–C and C–O stretching of the cellulose backbone near 1027 cm^−1^. As with the ester assignment, however, these bands overlap with the intrinsic absorptions of cellulose, and they are therefore interpreted as being consistent with the proposed interactions rather than as definitive proof.

### 3.8. Scanning Electron Microscopy

**Figure 9:**
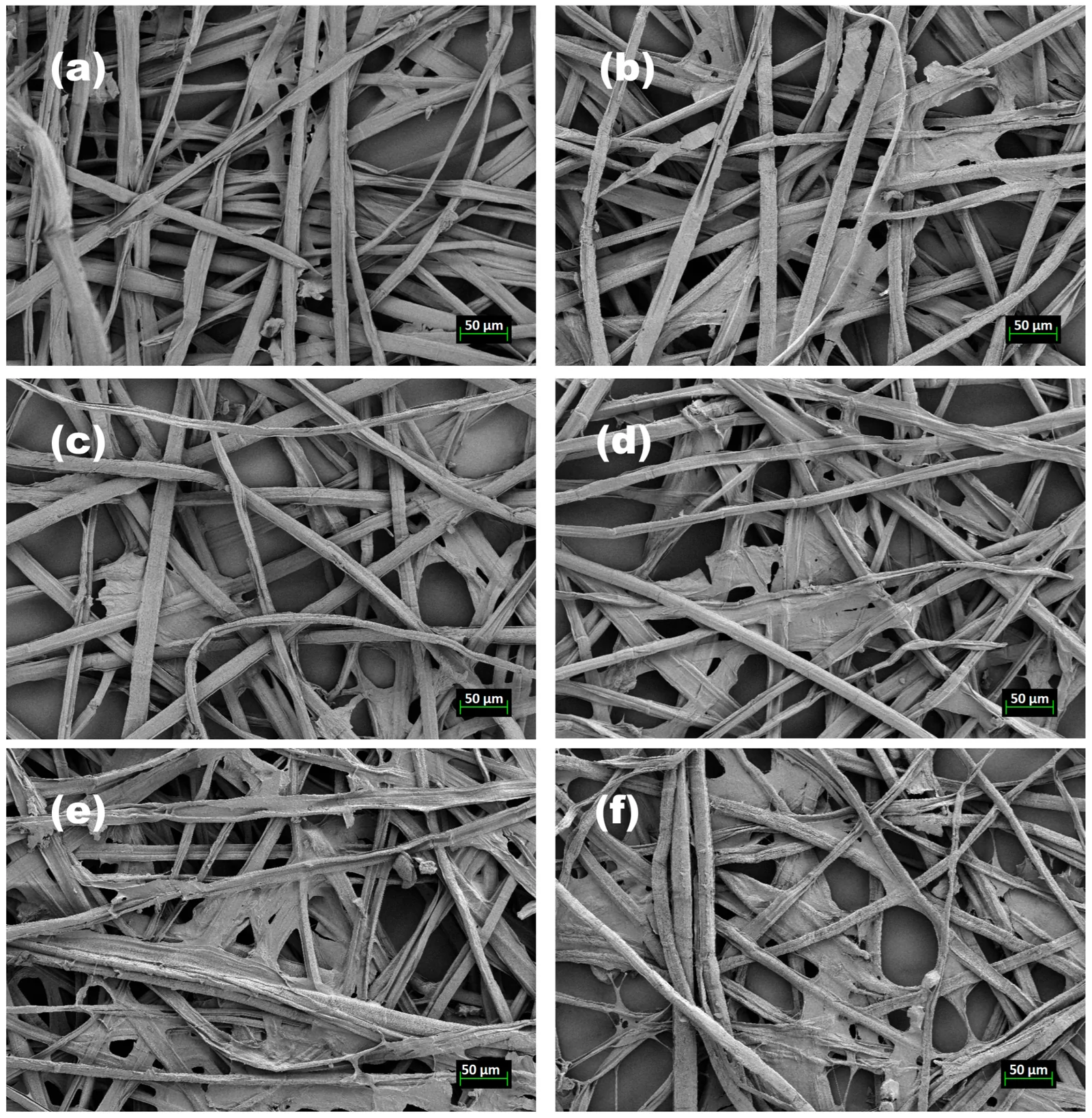
SEM micrographs of the ultra-lightweight Hanji fibers at 200× magnification: (**a**) untreated Hanji, (**b**) PAE-treated Hanji, (**c**) GPAM-treated Hanji, (**d**) PAE + CMC-treated Hanji, (**e**) GPAM + CMC-treated Hanji, and (**f**) the original MTP.

SEM can be used to examine the surface morphology and fiber structure of paper treated with wet-strength agents, including changes in the interfiber network and surface coverage [36,37]. Figure 9 shows SEM images of untreated ultra-lightweight Hanji and ultra-lightweight Hanji treated with wet-strength agents. In the untreated Hanji, the fibers were interwoven, and numerous pores were observed between the fibers. By contrast, in the Hanji treated with wet-strength agents, some interfiber pores appeared filled, and a dense film-like material, presumably originating from the applied agents, was observed between the fibers. The improvement in dry and wet strength may therefore be attributed to the coating of fiber surfaces and the filling of interfiber spaces by the wet-strength agents and CMC [38]. However, conventional surface SEM reveals morphological changes only; it cannot directly establish the penetration depth or the chemical identity of the material observed between the fibers. Confirming these aspects would require complementary techniques such as cross-sectional SEM or elemental mapping.

## 4. Conclusions

This study produced handmade ultra-lightweight Hanji using cooked traditional lye, following the traditional method for paper-based cultural heritage conservation, and improved water resistance by treating it with wet-strength agents PAE and GPAM, as well as the cellulose derivative CMC.

Accelerated aging tests were conducted to evaluate conservation suitability, aging stability, and the potential of handmade ultra-lightweight Hanji as a substitute for MTP, which is widely used in paper conservation.

The weight increase in handmade ultra-lightweight Hanji after the PAE treatment was higher than that after the GPAM treatment. Co-treatment with the cellulose derivative CMC further increased the weight uptake of the ultra-lightweight Hanji. Treatment with wet-strength agents also improved tensile strength, which was further enhanced when the paper was co-treated with CMC rather than treated with PAE or GPAM alone.

After accelerated aging, the strength measurement results showed that the dry tensile strength of the GPAM-treated handmade ultra-lightweight Hanji was superior to that of the PAE-treated Hanji. In addition, the *oebal* papermaking method, which uses the cross-fiber construction of handmade ultra-lightweight Hanji, resulted in a minimal difference in the wet tensile index between CD and LD fiber orientations. In contrast, MTP showed a clear difference in strength between the MD and CD. This directional stability is an important advantage of Hanji in conservation treatment.

After treatment with a wet-strength agent, the pH of the papers decreased further during accelerated aging. PAE-treated ultra-lightweight Hanji became strongly acidic, decreasing from an initial pH of 4.95 to 4.34–4.54 after aging, whereas GPAM-treated ultra-lightweight Hanji decreased from a near-neutral initial pH of 6.62 to a slightly acidic range of 5.52–5.68.

Because a less acidic pH is more favorable for the long-term conservation of cultural heritage materials, GPAM was considered more suitable than PAE as a wet-strength agent from the perspective of preservation and aging stability.

As the accelerated aging time increased during the accelerated aging of handmade ultra-lightweight Hanji treated with a wet-strength agent, the L* and a* values decreased, whereas the b* value increased. The observed decrease in L* was relatively small. Compared to GPAM treatment, PAE treatment resulted in a greater decrease in red tint and an increase in yellow tint. The change in color difference (ΔE) was more pronounced with CMC treatment, and co-treating the paper with CMC along with a wet-strength agent helped improve strength but negatively affected color stability.

Overall, this study aimed to develop a conservation material that combines traditionally produced handmade ultra-lightweight Hanji with modern papermaking additives, such as PAE and GPAM, to meet the requirements of low thickness, transparency, handling strength, and wet strength in paper conservation practice.

These results indicate that handmade ultra-lightweight Hanji, particularly when treated with GPAM, is promising for the conservation of paper-based cultural heritage, offering an alternative to MTP, which is currently the most widely used material.

This study focused on the fundamental material properties—mechanical performance, pH, and short-term accelerated-aging behavior—of wet-strength-treated handmade ultra-lightweight Hanji as a first step in evaluating its potential as an alternative to MTP. However, its performance as a conservation medium in practice, including application to real or model artifacts, reversibility and removability, interaction with original substrates and media, optical compatibility, and long-term stability, was beyond the scope of the present work, and is therefore an important subject for future research. In addition, future work will employ complementary surface-sensitive techniques, such as X-ray photoelectron spectroscopy (XPS) and variable-temperature FT-IR, to obtain more direct evidence of the bonding and hydrogen-bonding interactions between the wet-strength agents and cellulose.

## Figures and Tables

**Table 1:** Characteristics of the ultra-lightweight paper samples.

Sample	Cooking Chemical	Basis Weight (g/m^2^)	Apparent Density (g/cm^3^)	Forming Method	Origin
Ultra-lightweight Hanji	Plant-ash lye	7.2	0.30	Handmade (*Oebal choji*)	Korea
Machine-made thin paper (MTP)	Na_2_CO_3_	7.0	0.28	Machine-made	Japan

**Table 2:** Characteristics of the wet-strength agents.

Chemicals	Concentration (%)	Viscosity (cP)	pH	Product Code	Provider
PAE	As received	30.0	3.20	Fenno strengthPA 21	Kemira Co. (Helsinki, Finland)
	0.5 (applied)	6.0	3.30		
GPAM	As received	20.0	3.50	Fenno Rez 631 NC	
	0.5 (applied)	7.0	3.95		
CMC	As received	50–200	6.75	NF-9M40	NTPL (Sancheong, Republic of Korea)
	0.5 (applied)	16.6	6.50		

**Table 3:** Summary of ANOVA results (F, degrees of freedom (df), and *p*) for the effects of wet-strength agent, aging time, and testing direction on the tensile index, wet tensile index, and pH of ultra-lightweight Hanji.

Response/Factor	F	df	*p*
Dry tensile index—agent	503.1	1, 143	<0.001
Aging time	8.1	3, 143	<0.001
Direction	52.7	1, 143	<0.001
Agent × aging	1.2	3, 143	0.306
Agent × direction	75.6	1, 143	<0.001
Agent × aging × direction	16.3	3, 143	<0.001
Wet tensile index—agent	86.2	1, 65	<0.001
Agent × aging × direction	7.4	3, 65	<0.001
pH—agent	2617	1, 30	<0.001
Aging time	258.6	3, 30	<0.001

Note: Assumption checks: dry tensile—Shapiro–Wilk *p* = 0.195 (normal), Levene *p* = 0.021 (variance heterogeneity, reported); wet tensile—Shapiro *p* = 0.076, Levene *p* = 0.534 (both satisfied); pH—Shapiro *p* = 0.031 (marginal), Levene *p* = 0.732.

**Table 4:** Color parameters of the ultra-lightweight Hanji after accelerated aging. Values are expressed as mean ± standard deviation (SD).

Sample	Color	Aging Time (h)			
		0	24	48	72
Untreated	L*	80.48 ± 0.25	80.24 ± 0.26	80.14 ± 0.68	79.63 ± 0.64
	a*	−0.26 ± 0.01	−0.64 ± 0.01	−0.60 ± 0.05	−0.66 ± 0.01
	b*	4.94 ± 0.03	4.62 ± 0.17	2.90 ± 0.18	2.91 ± 0.06
	ΔE	—	0.55	2.09	2.23
PAE	L*	80.19 ± 0.23	79.56 ± 0.87	80.14 ± 0.65	80.10 ± 0.55
	a*	−0.38 ± 0.02	−0.96 ± 0.03	−1.03 ± 0.05	−1.05 ± 0.02
	b*	4.52 ± 0.16	5.83 ± 0.13	6.36 ± 0.34	6.57 ± 0.43
	ΔE	—	1.57	1.95	2.16
GPAM	L*	79.24 ± 0.26	79.07 ± 0.27	79.64 ± 0.22	79.02 ± 0.16
	a*	−0.39 ± 0.01	−0.59 ± 0.01	−0.61 ± 0.01	−0.59 ± 0.01
	b*	3.78 ± 0.08	4.58 ± 0.15	4.61 ± 0.07	4.81 ± 0.56
	ΔE	—	0.84	0.95	1.07
PAE + CMC	L*	58.38 ± 0.28	56.58 ± 1.90	56.10 ± 0.45	54.66 ± 0.30
	a*	−0.25 ± 0.02	−0.25 ± 0.02	−0.26 ± 0.01	−0.28 ± 0.02
	b*	0.73 ± 0.02	0.58 ± 0.13	0.77 ± 0.10	0.69 ± 0.06
	ΔE	—	1.80	2.27	3.72
GPAM + CMC	L*	57.84 ± 0.84	57.54 ± 0.45	56.09 ± 0.74	55.76 ± 0.13
	a*	−0.21 ± 0.02	−0.27 ± 0.01	−0.26 ± 0.01	−0.27 ± 0.01
	b*	0.68 ± 0.10	0.79 ± 0.07	0.95 ± 0.04	0.86 ± 0.04
	ΔE	—	0.33	1.77	2.09
MTP	L*	73.74 ± 0.43	73.28 ± 0.28	73.06 ± 0.32	74.49 ± 0.20
	a*	−0.33 ± 0.01	−0.33 ± 0.01	−0.35 ± 0.01	−0.37 ± 0.01
	b*	0.54 ± 0.04	0.68 ± 0.04	0.71 ± 0.07	0.85 ± 0.05
	ΔE	—	0.47	0.70	0.82

## Data Availability

The data presented in this study are available from the corresponding author upon reasonable request. The data are not publicly available because of potential proprietary concerns related to patent protection for this work.
